# Perceptions of Telehealth Physical Therapy Among Patients with Chronic Low Back Pain

**DOI:** 10.1089/tmr.2021.0028

**Published:** 2021-11-03

**Authors:** Julie M. Fritz, Elizabeth Lane, Kate I. Minick, Tyler Bardsley, Gerard Brennan, Stephen J. Hunter, Terrence McGee, Fenan S. Rassu, Stephen T. Wegener, Richard L. Skolasky

**Affiliations:** ^1^ Department of Physical Therapy and Athletic Training, University of Utah, Salt Lake City, Utah, USA.; ^2^ Rehabilitation Services, Intermountain Healthcare, Murray, Utah, USA.; ^3^ Division of Epidemiology, University of Utah School of Medicine, Salt Lake City, Utah, USA.; ^4^ Rehabilitation Therapy Services, Johns Hopkins Medicine, Lutherville, Maryland, USA.; ^5^ Department of Physical Medicine and Rehabilitation, The Johns Hopkins University School of Medicine, Baltimore, Maryland, USA.; ^6^ Department of Orthopaedic Surgery, The Johns Hopkins University School of Medicine, Baltimore, Maryland, USA.

**Keywords:** telehealth, back pain, rehabilitation, physical therapy

## Abstract

**Background:** Coronavirus disease 2019 prompted the rapid adoption of telehealth to provide physical therapy. Patients' perceptions about telehealth physical therapy are mostly unknown. This study describes perceptions of telehealth physical therapy among patients with chronic low back pain (LBP).

**Methods:** This study surveyed participants in an ongoing multisite clinical trial of nonpharmacological LBP treatments. Participants were asked about their willingness to use telehealth for physical therapy and with other providers and completed the PROMIS-29.

**Results:** Surveys were received from 102 participants (mean age = 48.5 [standard deviation; SD = 11.6]). Thirty-six (35.3%) expressed willingness to receive telehealth physical therapy, 22 were neutral (21.6%), and 44 were unwilling (43.1%). The percentage expressing willingness for telehealth physical therapy was lower than it was for family medicine (*p* < 0.001) or mental health (*p* < 0.001). Older (*p* = 0.049) and Black participants (*p* = 0.01) more likely expressed willingness to use telehealth for physical therapy.

**Conclusion:** Education and familiarity may help patients view telehealth physical therapy more favorably. Clinical Trial Registration (clinicaltrials.gov NCT03859713).

## Introduction

Chronic low back pain (LBP) is among the most common reasons for a health care visit in primary care and in physical therapy.^1,2^ Guidelines emphasize nonpharmacological treatment, yet uptake of this recommendation in practice is inconsistent, and overuse of opioid therapy persists.^3,4^ In the United States, telehealth delivery of nonpharmacological treatments including physical therapy was uncommon before the onset of coronavirus disease 2019 (COVID-19).^5^ Increasing the uptake of nonpharmacological treatments requires attention to factors impacting their transition to telehealth delivery.

COVID-19 prompted rapid acceleration of telehealth delivery across all sectors of health care including physical therapy.^6,7^ The rapidity of this transition did not permit lengthy consideration of public perceptions or willingness to participate in telehealth delivery of specific services. Considering the low utilization before COVID-19, it is unclear whether patients would consider telehealth physical therapy equivalent to in-person care.^8^ If patients with LBP do not perceive telehealth physical therapy as a viable option, it may adversely impact the likelihood of compliance with this nonpharmacological treatment strategy.

The purpose of this study is to describe the perceptions of telehealth delivery of physical therapy among patients with chronic LBP. We surveyed participants who previously enrolled in a clinical trial investigating nonpharmacological treatments for chronic LBP. Specifically, we surveyed participants to describe their willingness to attend telehealth physical therapy for chronic LBP, compare the willingness to attend telehealth physical therapy with the willingness to see other providers through telehealth, and to compare patient factors between those with varying levels of willingness to participate in telehealth physical therapy.

## Methods

This cross-sectional survey study recruited participants previously enrolled in a clinical trial examining nonpharmacological treatments (physical therapy, cognitive behavioral therapy, and mindfulness) for chronic LBP. Details of the parent trial (clinicaltrials.gov NCT03859713) are published.^9^ The trial was approved by the University of Utah Institutional Review Board (IRB) acting as the single IRB. Participating sites were the University of Utah and Intermountain Healthcare based in Salt Lake City, Utah, and Johns Hopkins University based in Baltimore, Maryland.

### Participants

Participants were adults, aged 18–64 years, with nonspecific chronic LBP with at least moderate pain (≥4 on a 0–10 numeric pain rating scale) and LBP-related disability (≥24 on the Oswestry Disability Index). Chronic LBP was defined using published criteria as LBP being a problem for at least 3 months, and an ongoing problem “almost every day” or “everyday” for the past 6 months.^10^ Exclusion criteria included red flags suggesting a potentially serious cause of LBP (e.g., neoplasm and osteomyelitis), non-English speaking, having received one of the study interventions for LBP in the past 90 days, and lumbar spine surgery in the past year.^9^

### Study procedures

The parent clinical trial enrolled 181 participants from April 2019 through March 2020. COVID-19 restrictions prompted suspension of enrollment in March 2020. Participants enrolled before suspension were invited to complete the survey between September 1 and October 15, 2020. Participants received $25 for completing the survey online or over the telephone with study personnel.

### Measures

Demographic information was collected at the time of enrollment to the parent study. Additional data collected at the time of survey included experience with telehealth for health care visits before and after the onset of COVID-19, and barriers to using telehealth including access to technology and the internet, and availability of a quiet space without interruptions in the home for telehealth visits. The PROMIS-29 v2.0 was completed to assess participants' self-report health status. The PROMIS-29 assesses pain intensity using a single 0–10 numeric rating and seven health domains (physical function, fatigue, pain interference, depressive symptoms, anxiety, ability to participate in social roles, and sleep disturbance) using four items for each domain.^11^ Health domain scores are converted to T-scores with mean = 50 and standard deviation (SD) = 10. Higher scores indicate a greater presence of the quantity assessed.

To examine participants' perceptions of telehealth, we collected willingness to use telehealth for various types of provider visits. Participants were asked “How willing would you be to use telehealth to see the following type of health care provider?” Response options *presented to the participant* were collected using a 5-point scale of “Very Unwilling,” “Somewhat Unwilling,” “Neutral,” “Somewhat Willing,” and “Very Willing.” Participants were asked about willingness to see a physical therapist, as well as a mental health therapist, family doctor, or urgent care provider.

### Data analysis

We divided respondents based on willingness to use telehealth physical therapy into three categories: “Unwilling” (including patients who were somewhat or very unwilling), “Neutral,” or “Willing” (including patients who were somewhat or very willing). Participant characteristics were calculated for the cohort and for each category of willingness to use telehealth physical therapy. Willingness to use telehealth for other providers was reported using the same three categories. We compared participant variables across categories of willingness to use telehealth physical therapy using chi-square and fishers exact test for categorical or nominal level data, and analysis of variance (ANOVA) or Kruskal Wallis test for continuous level data. The Friedman test was used to test for a difference in willingness across provider types, and pairwise comparisons with physical therapy were tested with the Wilcoxon Signed Rank Sum test. Owing to the exploratory nature of this analysis, we did not use a multiple comparisons adjustment. We carried out all analyses in SAS statistical software version 9.4 (SAS Institute, Inc., Cary, NC, USA).

## Results

A total of 157 participants were contacted for participation, of whom 104 (66.2%) completed the survey. The question regarding willingness to use telehealth physical therapy was not completed by two respondents, leaving data from 102 participants for analysis. Descriptive characteristics are provided in Table 1. Fifty-seven participants (55.8%) were recruited from Baltimore, and 45 (44.2%) participants from Salt Lake City. Most participants (*n* = 79, 77.4%) had used telehealth for a health care visit, only 5 of these 79 individuals (6.3%) indicated using telehealth before COVID-19. Few participants noted barriers to telehealth. Nine participants (8.9% of 101 respondents) indicated uncertainty about access to technology (internet or internet-enabled devices) and 20 participants (19.8% of 101 respondents) indicated uncertainty about their ability to find space without interruptions in their home for telehealth sessions.

**Table 1.** Participant Characteristics for the Entire Sample and by Willingness to Use Telehealth Physical Therapy

Characteristic	All participants (*N* = 102)	Willingness to attend PT telehealth visit			*p*-Value
		Unwilling (*n* = 44)	Neutral (*n* = 22)	Willing (*n* = 36)	
Age^a^ (mean, SD)	48.5 (11.6)	45.9 (12.1)	47.5 (12.8)	52.2 (9.6)	0.049^*^
Gender^a^ (*n*, % female)	71 (69.6)	29 (65.9)	18 (81.8)	24 (66.7)	0.37^‡^
Body mass index^a^ (mean, SD)	33.0 (9.01)	32.5 (8.94)	32.0 (8.60)	34.1 (9.50)	0.68^*^
Current smoker^a^ (*n*, % yes)	20 (19.6)	8 (18.2)	5 (22.7)	7 (19.4)	0.91^‡^
Ethnicity^a^ (*n*, % Hispanic/Latino) (*n* = 101)	9 (8.9)	3 (7.0)	3 (13.6)	3 (8.3)	0.61§
Race^a^ (*n* = 100)					
Black or African American (*n*, %)	36 (36)	17 (39.5)	1 (4.5)	18 (51.4)	0.010^‡^
White (*n*, %)	57 (57)	23 (53.5)	19 (86.4)	15 (42.9)	
Other (*n*, %)	7 (7)	3 (7)	2 (9.1)	2 (5.7)	
Highest education level^a^					
Completed college degree (*n*, %)	44 (43.1)	22 (50.0)	10 (45.5)	12 (33.3)	0.18§
Completed high school (*n*, %)	48 (47.1)	16 (36.4)	12 (54.5)	20 (55.6)	
Did not complete high school (*n*, %)	10 (9.8)	6 (13.6)	0 (0)	4 (11.1)	
Time from first LBP episode^a^					
1 year or less (*n*, %)	13 (12.8)	7 (15.9)	4 (18.2)	2 (5.6)	0.18§
2–5 years (*n*, %)	28 (27.4)	8 (18.2)	8 (36.4)	12 (33.3)	
>5 years (*n*, %)	61 (59.8)	29 (65.9)	10 (45.4)	22 (61.1)	
Has had a health care visit using telehealth (*n*, % yes)	79 (77.4)	32 (72.7)	17 (77.2)	30 (83.4)	0.52^‡^
Has a technology concern for using telehealth (*n*, % yes) (*n* = 101)	9 (8.9)	4 (9.1)	2 (9.5)	3 (8.3)	0.99§
Has a concern about having quiet space for telehealth	20 (19.8)	8 (18.2)	7 (33.3)	5 (13.9)	0.19^‡^

*p*-Values compare willingness categories using the appropriate statistical test (^*^analysis of variance, ^‡^chi squared test, §Fishers exact).

^a^ Indicates data taken from variables collected at the time of enrollment in the parent randomized clinical trial.

LBP, low back pain; PT, physical therapy.

Overall, 36 participants (35.3%) indicated a willingness to use telehealth physical therapy, 22 (21.6%) were neutral, and 44 (43.1%) indicated they were unwilling to use telehealth physical therapy. Participants willing to use telehealth physical therapy were older (*p* = 0.049) and more likely to be Black (*p* = 0.018) (Table 1). Other variables did not differ between categories of willingness to use telehealth physical therapy.

Participants' health status at the time of survey completion is reported in Table 2. Overall, participants reported levels of physical function and pain interference that were 1 SD below population averages, indicating an impact of pain on daily activities. There were no differences in health domains across categories of willingness to use telehealth physical therapy.

**Table 2.** Participant Health Characteristics for the Entire Sample and by Willingness to Use Telehealth Physical Therapy as Assessed Using the PROMIS-29 v.2

Health domain	All participants (*N* = 102)	Willingness to attend PT telehealth visit			*p*-Value
		Unwilling (*n* = 44)	Neutral (*n* = 22)	Willing (*n* = 36)	
Anxiety	55.8 (40.3, 63.4)	55.8 (40.3, 59.5)	57.7 (48, 71.2)	57.7 (40.3, 63.4)	0.30
Physical function	39.1 (35.6, 45.3)	39.1 (35.6, 48)	40.4 (35.6, 48)	39.1 (33.3, 45.3)	0.57
Depression	51.8 (41, 59.7)	51.8 (41, 58.1)	56.4 (41, 67.5)	51.8 (41, 61.4)	0.35
Fatigue	57.0 (48.6, 62.7)	56.1 (48.6, 62.7)	57.0 (48.6, 64.6)	55.1 (47.3, 62.7)	0.54
Sleep disturbance	56.1 (50.5, 61.7)	56.1 (52.4, 61.7)	55.2 (47.3, 62.8)	56.1 (47.3, 61.7)	0.74
Social role participation	44.2 (40.5, 51.9)	44.2 (42.3, 51.9)	44.2 (37.3, 51.9)	44.2 (40.5, 51)	0.80
Pain interference	61.2 (55.6, 66.6)	61.2 (55.6, 66.6)	61.2 (53.9, 69.7)	61.2 (55.6, 65.9)	0.99
Pain intensity (mean, SD)	5.4 (2.7)	5.4 (2.8)	4.9 (3.1)	5.7 (2.6)	0.47

Higher scores represent a greater presence of the domain being evaluated. Values represent median score with interquartile range unless otherwise indicated (*p*-values compare willingness categories using a Kruskal Wallis test with the exception of pain intensity that used an analysis of variance).

**FIG. 1.**
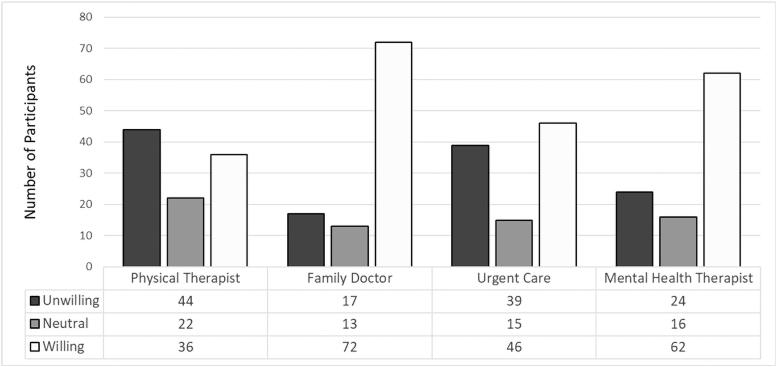
Willingness to use telehealth by provider type.

Participants' willingness to use telehealth for other health care providers is shown in Figure 1. The overall distribution of willingness differed across provider types (*p* < 0.001). Participants were less likely to be willing to use telehealth for physical therapy (35.3%) than they were for a visit with a family doctor (70.6%, *p* < 0.001) or mental health therapist (60.8%, *p* = 0.001). Willingness did not differ between physical therapy and urgent care (46.0%, *p* = 0.084).

## Discussion

Our objective was to describe perceptions of telehealth delivery of physical therapy among patients with chronic LBP and to explore factors associated with these perceptions. We found that willingness to use telehealth for physical therapy was low (36.1%), and significantly lower than the willingness to use telehealth with other providers. Respondents' physical and psychological health domains were not associated with willingness to use telehealth physical therapy. Willingness was related to age and race, with older and Black participants more likely to express willingness to use telehealth physical therapy.

Lower willingness to use telehealth physical therapy for persons with chronic LBP is not a surprising finding. Before COVID-19, there was little utilization of telehealth physical therapy in the United States because of restrictions on reimbursement by most payers including Medicare. Modifications and waivers were granted in response to COVID-19,^12^ yet even after these changes, use of telehealth physical therapy has been low. For example, Werneke and colleagues reported on >220,000 episodes of care in outpatient therapy settings nationwide and found only 5–10% included telehealth in the second and third quarter of 2020.^6^ National studies indicate that the uptake of telehealth for behavioral health and primary care has been much greater.^5^ Limited experience with telehealth physical therapy both before and after the onset of COVID-19 may contribute to perceptions that telehealth is a less viable option for physical therapy.

Reasons for lower willingness to use telehealth physical therapy may relate to the use of physical touch, which is fundamental to physical therapy care for chronic LBP, but is not a central component of care in family medicine or behavioral health settings. For example, a recent survey of public perceptions of telehealth visits for orthopedic care found that 81% of respondents agreed that the lack of physical touch was a concern.^13^ A survey of nonpharmacological pain providers, most of whom were physical therapists, also found that many believed the absence of touch when using telehealth hampers effective diagnosis and treatment.^14^ It is likely that concerns about the lack of touch in telehealth delivery influenced participant responses to our survey, many of whom had experience receiving in-person physical therapy for their LBP.

Hands-on interventions are effective for patients with chronic LBP,^15^ however, effect sizes are small and some guidelines consider hands-on therapies such as manipulation and massage as adjunctive treatments for chronic LBP.^3^ Exercise, advise, and education are first-line interventions for chronic LBP,^3,16^ and these treatments are also more readily adaptable to telehealth delivery, suggesting the potential for telehealth physical therapy to be effectively adapted to telehealth, despite perceptions of physical therapists and patients. Emerging studies examining physical therapy provided using telehealth suggest that outcomes are equivalent to in-person care,^6,17^ and future protocols may make greater use of hybrid in-person and telehealth approaches to allow for physical contact while also making use of remote technologies. Much of the existing evidence supporting telehealth physical therapy used hybrid approaches,^18^ and this approach may be more satisfactory to patients.^19^

There is optimism that greater adoption of telehealth delivery can help alleviate disparities.^20^ A survey on willingness to use telehealth conducted before COVID-19, however, reported less willingness among older and Black respondents.^21^ Earlier reports after the onset of COVID-19 raised concerns that older and Black patients were less likely to use telehealth.^22–24^ In our survey we found that older and Black respondents were more likely to express willingness to use telehealth physical therapy. The contradictory results from our survey may reflect changes in attitudes toward telehealth in response to COVID-19-related disruptions, as our survey was conducted during September to October of 2020. Also, the upper age limit of respondents in our survey was 64 years, thus our sample did not include elderly individuals. Our findings may also reflect differences between intentions and barriers that arise when attempts are made to actually access care. Attention to equity in access to telehealth physical therapy is an important ongoing consideration given the well-established disparities that exist in pain management.^25,26^

### Limitations

Our sample included a limited number of participants from three health care systems in two geographic regions, and thus is not nationally representative. Participants in our survey had previously enrolled in a clinical trial examining interventions for chronic LBP including physical therapy. Although the trial intervention period was completed for all respondents, participation in the trial may have influenced responses.

## Conclusions

It is likely that telehealth delivery of physical therapy for chronic LBP will be more prevalent post-COVID-19. The results of this study suggest that patient perceptions and willingness to use telehealth physical therapy for chronic LBP may be lower than for care from other providers. Further education may be necessary to help patients understand the potential benefits of physical therapy for LBP provided by telehealth. Attention to the design and implementation of telehealth delivery of physical therapy is needed to educate patients and address concerns they may have about receiving telehealth.

## Abbreviations Used

COVID-19: coronavirus disease 2019
IRB: institutional review board
LBP: low back pain
SD: standard deviation
